# Tumor Microenvironment Remodeling in Gastrointestinal Cancer: Role of miRNAs as Biomarkers of Tumor Invasion

**DOI:** 10.3390/biomedicines11061761

**Published:** 2023-06-19

**Authors:** Valeria Lucarini, Daniela Nardozi, Valentina Angiolini, Monica Benvenuto, Chiara Focaccetti, Raffaele Carrano, Zein Mersini Besharat, Roberto Bei, Laura Masuelli

**Affiliations:** 1 Department of Experimental Medicine, University of Rome “Sapienza”, Viale Regina Elena 324, 00161 Rome, Italy; valeria.lucarini@uniroma1.it (V.L.); valentina.angiolini@uniroma1.it (V.A.); zeinmersini.besharat@uniroma1.it (Z.M.B.); 2 Department of Clinical Sciences and Translational Medicine, University of Rome “Tor Vergata”, Via Montpellier 1, 00133 Rome, Italy; daniela.nardozi@uniroma2.it (D.N.); monica.benvenuto@unicamillus.org (M.B.); chiara.focaccetti@uniroma2.it (C.F.); raffo9318@gmail.com (R.C.); bei@med.uniroma2.it (R.B.); 3 Departmental Faculty of Medicine and Surgery, Saint Camillus International University of Health and Medical Sciences, via di Sant’Alessandro 8, 00131 Rome, Italy

**Keywords:** gastrointestinal tumors, miRNA, biomarker, nutraceuticals, tumor microenvironment, tumor cell migration, metastasis, EMT

## Abstract

Gastrointestinal (GI) cancers are the most frequent neoplasm, responsible for half of all cancer-related deaths. Metastasis is the leading cause of death from GI cancer; thus, studying the processes that regulate cancer cell migration is of paramount importance for the development of new therapeutic strategies. In this review, we summarize the mechanisms adopted by cancer cells to promote cell migration and the subsequent metastasis formation by highlighting the key role that tumor microenvironment components play in deregulating cellular pathways involved in these processes. We, therefore, provide an overview of the role of different microRNAs in promoting tumor metastasis and their role as potential biomarkers for the prognosis, monitoring, and diagnosis of GI cancer patients. Finally, we relate the possible use of nutraceuticals as a new strategy for targeting numerous microRNAs and different pathways involved in GI tumor invasiveness.

## 1. Introduction

Gastrointestinal (GI) cancers are complex and heterogeneous diseases caused by the interaction of genetic and environmental factors. These types of tumors can affect several organs, such as the pancreas, liver, gallbladder, bile ducts, and colon. Because of the large cell mass and rapid turnover, GI cancers are among the most frequent neoplasms and are responsible for about half of all cancer-related deaths [1]. In fact, they account for 26% of global cancer incidence and 35% of all cancer-related deaths [2]. These neoplasms have different clinical features but share some similar characteristics; the most common is their poor prognosis meaning that most treatments can often be ineffective. Although much progress has been made in the early detection of colorectal cancer (CRC) through screening, the prognosis of other GI tumors tends to be unfavorable due to patients presenting late-stage diagnoses [3]. Current therapies involve adjuvant and neoadjuvant chemoradiotherapy, targeted therapy, or immunotherapy, which stimulate an antitumor immune response [3,4]. Despite these multidisciplinary approaches, a fair number of patients still develop distant metastasis and show resistance to therapy [5,6]. Indeed, metastasis is the leading cause of death from GI cancer, so it is of paramount importance to study the players and mechanisms that regulate tumor migration, invasion, and metastatic dissemination, in order to evaluate the possibility of using these substrates as new therapeutic targets.

In this review, we first provide an overview of the molecular basis of the tumor microenvironment (TME) in regulating cell migration, invasion, and metastasis formation in four GI tumors: gastric cancer (GC), cholangiocarcinoma (CCA), hepatocellular carcinoma (HCC), and CRC. We also describe the role of miRNAs as tumor invasivity biomarkers with a final focus on nutraceuticals in driving TME remodeling.

## 2. Gastrointestinal Cancers: A General Overview

### 2.1. Gastric Cancer

GC is one of the most common malignancies in the world, accounting for 5.6% of all cancers and 7.7% of all cancer-related deaths worldwide [7]. GC can develop in the stomach and spread throughout other organs, such as the small intestines, lymph nodes, liver, pancreas, and colon [8]. Most GCs are adenocarcinomas, which can be further subdivided into intestinal and diffuse types according to the Lauren classification [9,10] and are also associated with infectious agents, including the bacterium Helicobacter pylori and the Epstein–Barr virus [11,12]. Therapies are mainly based on chemotherapy, radiotherapy, and immunotherapy and are effective alternatives for patients who cannot undergo surgical resection [8,13,14]. However, a proportion of patients can develop resistance to therapy with subsequent metastasis formation. About 40% of patients with GC present metastasis, and only 5% of these patients have a 5-year survival [15]. The reasons are complex, and one of the most important issues is the potentiality of cancer cells to metastasize. The prognosis of GC patients with metastatic disease remains poor due to a lack of effective therapies and limited information regarding the mechanisms that regulate metastasis formation [16]. TME plays a key role in the initiation and formation of pre-metastatic niches, promoting the ability of cancer cells to proliferate, invade, and migrate [17]. Indeed, TME regulates multiple signaling pathways involved in migration and invasiveness, such as the Wnt/β-catenin pathway, nuclear factor kappaB (NF-κB), extracellular regulated kinase 1/2 (ERK1/2), transforming growth factor-β (TGF-β)/Smad, and phosphatidylinositol 3-kinase (PI3K)/Akt pathway [5,17].

### 2.2. Cholangiocarcinoma

CCA includes a heterogeneous group of malignancies arising along the biliary tree epithelium and represents an estimated 3–5% of all GI system malignancies [18,19]. According to their anatomical origin, CCAs are classified into three groups: intrahepatic (10% of all CCA), perihilar (50–60%), or distal (20–30%) [19]. They share similarities but also have important differences that can affect the pathogenesis and outcome. Although this anatomical classification is widely used, other factors such as tumor growth pattern and cell of origin (cholangiocytes, perishable glands, liver progenitor cells, or hepatocytes) provide alternative methods of classification that can better predict tumor behavior [20]. Patients with CCA are often asymptomatic in the early stages of the tumor, and, for most of them, diagnosis occurs at an advanced stage. Both late diagnosis and high chemoresistance of these tumors compromise possible curative treatment options and contribute to their poor prognosis [19]. Currently, the most effective treatment options consist of surgery or liver transplantation, although these are indicated in less than 30% of patients, and the possibility of tumor recurrence is high [21]. For unresectable cases, palliative treatment, consisting of a combination of different chemotherapeutic agents, such as gemcitabine and cisplatin, and the use of immune checkpoint inhibitors (ICIs), remains the only possible option, with overall survival of 12 months [21]. Similar to GC in CCA, TME plays a primary role in the ability of tumor cell invasion and subsequent metastasis formation. Indeed, the increase in TGF-β levels in CCA is responsible for the switch of E-cadherin to N-cadherin, which leads to a subsequent loss of cell–cell adhesion and promotion of tumor invasion [22,23]. In addition, cancer-associated fibroblasts (CAFs) in the adjacent stroma release factors responsible for invasion and metastasis formation through the E-to-N-cadherin conversion and activation of the PI3K-Akt pathway [22,24].

### 2.3. Hepatocellular Carcinoma

Hepatic carcinoma (HCC) is the most frequent neoplasm of the liver. It originates from the malignant transformation of hepatocytes and frequently evolves from chronic diseases such as hepatitis, fibrosis, and cirrhosis [25]. HCC is the third leading cause of cancer deaths worldwide, with a relative 5-year survival rate of approximately 18%. The similarity between incidence and mortality underlines the dismal prognosis associated with this disease [26]. When diagnosed at an early stage, it is curable with localized approaches, including surgical resection, chemotherapy, radiation therapy, or liver transplantation [27,28]. However, HCC is usually diagnosed in advanced stages when the tumor is unresectable, rendering these treatments ineffective. Hepatic tumor development is controlled by both extracellular factors and intracellular signaling pathways [29]. These pathways are found to be significantly altered and upregulated to promote tumorigenesis and cancer progression. TGF-β plays an important role in HCC tumor progression because it promotes epithelial–mesenchymal transition (EMT) and, thus, tumor cell motility [30]. It is worth noting that along with the role of TGF-β, other signaling pathways such as PI3K/Akt, ERK/c-Jun amino-terminal kinases (JNK), Wnt/β-catenin, and NF-κB are also closely involved in the migration and invasiveness of HCC cancer cells and metastasis formation [29].

### 2.4. Colorectal Cancer

CRC is the second most common cancer in terms of mortality and the third type in frequency in both men and women [7,31]. About 50% of patients develop early metastasis and a poor prognosis because of resistance to chemotherapy [7,32]. This is due to the different molecular mechanisms responsible for CRC tumor progression. From a genomic standpoint, CRC is not a single disease but a heterogeneous group of malignancies arising within the colon. Indeed, the accumulation of genetic and epigenetic alterations deregulates several signaling pathways responsible for the activation of proliferative signaling, resistance to cell death, escape from immunosurveillance, deregulation of energy metabolism, induction of angiogenesis, and tumor invasion [33]. Approximately 15% of CRC cases display microsatellite instability secondary to a defective mismatch repair [34]. Based on this, CRC may have alternative therapeutic options relying on the administration of certain immunologic agents. Actually, immunotherapy is widely used for the treatment of CRC, along with preoperative radiation therapy, surgery, chemotherapy, and targeted therapy [35,36,37]. Additionally, in CRC, crosstalk between TME and tumor cells plays a key role in invasiveness, metastasis formation, and drug resistance. TME regulates a plethora of cellular signaling pathways that control multiple stages of tumor progression and invasion, such as mitogen-activated protein kinase (MAPKs), PI3K/Akt, NF-κB, JAK/STAT, Wnt/β-catenin, TGF-β, and Notch [38].

### 2.5. Gastrointestinal Stromal Tumor

Gastrointestinal stromal tumors (GISTs) are a subgroup of rare mesenchymal GI tumors that arise most frequently in the stomach (~60%) and small intestine (~25%) and less frequently in the rectum (~5%) and esophagus (<1%) [39,40]. Recently, the incidence has increased with 10–15 new cases per 100,000/year, mainly in older patients and rarely in younger patients [41,42]. GISTs develop from a particular type of cell called interstitial cells of Cajal (ICC). These are “pacemaker” cells responsible for the physiological contraction of the digestive tract and have specific characteristics according to their localization in the GI tract [43]. ICC and smooth muscle cells derive from the same precursors and express the tyrosine kinase receptor (KIT). While mature smooth muscle cells lose KIT expression, ICCs continue to express it [44]. In GISTs, diffuse hyperplasia of ICC is observed, which is considered a pre-neoplastic lesion. The pathogenesis of GISTs is determined by mutations in KIT or platelet-derived growth factor receptor A (PDGFRA) genes [45]. Surgery is the primary choice for resectable GISTs; however, the therapeutic treatment for unresectable metastatic GIST patients has been radically changed by the introduction of targeted therapy [46]. Administration of imatinib mesylate, together with other kinase inhibitors, has been associated with good improvement in clinical outcomes and survival [46]. Despite the many benefits, patients may develop resistance due to secondary mutations in KIT and PDGFRA that lead to the deregulation of crucial signaling pathways involved in cell proliferation and migration, such as RAS/RAF/MAPK and PI3K/Akt/mTOR [47].

## 3. Tumor Microenvironment in Promoting Cancer Cells Migration and Invasion

**Figure 1:**
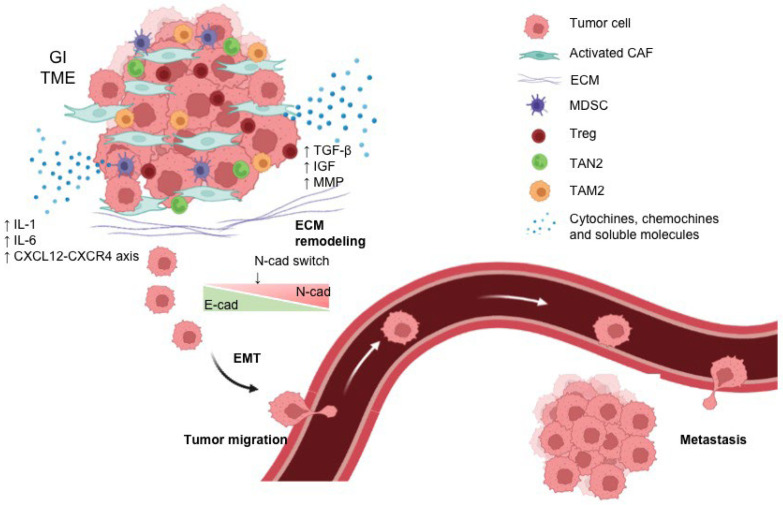
Schematic representation of gastrointestinal TME and biological events leading to cell migration and metastasis formation. A pro-tumoral environment is established in the TME of GI tumors given by the presence of activated CAFs and immunosuppressive immune cells, leading to the production of cytokines, chemokines, and soluble factors. These are responsible for ECM remodeling and promotion of EMT that enhances tumor cell motility and subsequent metastasis formation. The figure was created with BioRender.com.

Over the past decade, several studies have shown that cancer growth is determined not only by tumor cells but also by the TME [48]. Indeed, it consists of a complex network of malignant and non-malignant cells that potentiate cancer progression and metastasis and modulate responses to therapy [48,49]. Specifically, the TME is composed of various elements, including tumor cells, immune cells such as T cells, B cells, dendritic cells (DC), myeloid-derived suppressor cells (MDSCs), tumor-associated macrophages (TAMs), CAFs, and blood and lymphatic vessels [50,51]. Intercellular communication is driven by a complex and dynamic network of cytokines, chemokines, growth factors, and inflammatory and matrix-remodeling enzymes, all of which can promote tumor progression and invasion (Figure 1) [52]. In particular, the TME of GI tumors is characterized by the presence of CAFs, which promote tumor growth, angiogenesis, invasion, and metastasis, along with extracellular matrix (ECM) remodeling and even chemoresistance [53]. In addition, TME is defined by the presence of an immunosuppressive immune infiltrate mainly composed of M2 macrophages, MDSCs, and T regulatory cells (Tregs), and finally by the presence of soluble factors such as TGF-β, which has a primary role in promoting tumor migration and metastases (Figure 1) [54].

### 3.1. Cancer-Associated Fibroblasts

CAFs constitute a dominant stromal component of the TME of GI cancers and contribute in many ways to tumor progression and resistance to therapy [53,55,56]. They originate predominantly from tissue-resident fibroblasts that are activated in response to signals from cancer cells and the TME [57]. In GI tumors, particularly in CRC and HCC, the presence of activated CAFs lead to increased expression of activated markers, such as α-smooth muscle actin (α-SMA) and fibroblast activation protein (FAP), and the production of large amounts of glycoproteins, including tenascin-C and collagen maturation enzymes, which are responsible for ECM remodeling [58,59]. Alterations in the biomechanical properties of the ECM can trigger cancer cell migration [53,60]. CAFs can generate gaps in stromal and basement membrane components that are connected through cell–cell junctions to promote EMT and mediate collective migration of tumor cells through matrix metalloproteases (MMP)-dependent or -independent mechanisms [61]. In addition, CAFs produce interleukin-(IL)-6 that induces the activation of the JAK2/STAT3 axis in GC and promotes EMT, increasing migration and invasion [62].

Activated CAFs can even produce abundant soluble molecules, including basic fibroblast growth factor (bFGF), members of the vascular endothelial growth factor (VEGF) family, platelet-derived growth factor (PDGF), ligands of epidermal growth factor receptor (EGFR), interleukins, and TGF-β [63,64]. The production of these soluble factors promotes cancer invasion and metastasis formation [63]. Further studies have shown that CXCL12 and its receptor CXCR4 derived from CAFs can promote cell invasion in GC and CRC tumors [65]. Activation of the CXCL12/CXCR4 axis in the TME, thus, provides paracrine signaling that mediates integrin β1 clustering on the surface of tumor cells, promoting tumor EMT [65]. In addition, CAFs recruit and polarize immune cells such as macrophages, neutrophils, T lymphocytes, and DCs toward a pro-tumorigenic phenotype by secreting various cytokines, chemokines, and other effector molecules such as IL-6, IL-8, CXCL12, CCL2, TGF-β, SDF-1, VEGF, and indoleamine-pyrrole2,3-dioxygenase (IDO) [66,67,68,69].

Finally, the accumulation of activated CAFs correlates with resistance to therapy. For example, the increase in FAP protein can cause resistance to chemotherapy, radiotherapy, and immune checkpoint blockade [55,70]. Furthermore, in experimental models of CRC, chemotherapy-stimulated CAFs increase the secretion of specific cytokines, such as IL-17A, which promotes chemoresistance through the NF-κB pathway and increases tumor invasion and growth in vivo. Of note, an increase in IL-17 was also observed in patients with therapy-resistant CRC [55,71].

### 3.2. Cytokines and Chemokines

Cytokines and chemokines are inflammatory mediators secreted by several components of the TME. Their action in promoting cancer development includes an antagonizing antitumor immune response, recruiting tumor-supportive stromal cells and immune-suppressive cells, inducing angiogenesis and metastasis, and altering the responses to therapeutic agents [72].

Several cytokines may contribute to tumor progression, particularly IL-1β, TNFα, and IL-6 play key roles in promoting and enhancing EMT. IL-6, secreted by CAFs, promotes STAT3 activation and, thus, tumor invasion and metastasis formation [73]. Similar to IL-6, IL-23 also acts by increasing the expression level of STAT3 and inducing EMT-mediated metastasis [74].

In addition to cytokines, several chemokines are also responsible for promoting tumor invasiveness. CXCL1 promotes the proliferation and migration of colon cancer cells and has a facilitating effect on tumor angiogenesis [75]. Increased CXCL1 promotes TAM2 migration by inhibiting the recall of CD4+ and CD8+ T cells at tumor sites [76]. CXCL5, in the GC TME, facilitates metastasis by promoting the invasion and migration of tumor cells through the activation of the ERK signaling pathway in cancer cells [77,78]. The CXCL12–CXCR4 axis is particularly important in tumor development to participate in CRC, pancreatic cancer, and HCC metastasis [79,80]. The binding of CXCL12 to its receptor CXCR4 on CRC cells causes pro-metastatic signaling through decreasing E-cadherin and inducing ICAM-1 expression [79]. CXCL12–CXCR4 also induces the Wnt/β-catenin pathway with increased MMP-2, MMP-9, and plasminogen activator and consecutive metastatic initiation in CRC [81]. As mentioned above, CAFs are responsible for the production of CXCL12; several studies have claimed that CXCL12 increases the proliferation, migration, and invasion of CRC cells through the induction of M2 polarization [65]. Another chemokine associated with the development of CRC and its liver metastasis is CXCL8, which promotes angiogenesis, proliferation, invasion, migration, and survival of tumor cells through the induction of EMT [82]. TGF-β promotes the generation of an immunosuppressive environment through the recall of TAM2, MDSC, and Tregs at the tumor site via activation of the Smad transcription factor [83,84,85,86]. TGF-β is a dominant effector in all GI cancers and mediates the conversion of fibroblasts to CAFs, promoting cell migration and tumor invasiveness through the induction of EMT [64,85]. In these tumors, TGF-β may have an antitumoral function in the early stage of tumor development by inducing apoptosis; on the contrary, in more advanced stages, it can support tumor progression by improving cell survival, EMT, migration, invasion, and metastasis [85]. In addition, TGF-β induces MMP-8 gene expression through PI3K/Akt/Rac1 signaling in HCC cells that promotes tumor cell EMT and malignant progression [87]. Finally, in addition to TGF-β, insulin-like growth factor-1 (IGF-1) can also promote cancer proliferation and survival, inducing EMT that contributes to tumor migration, invasiveness, and metastasis [88].

### 3.3. Immune Infiltrating Cells

The immune cell populations within the TME play a significant role in patient prognosis and response to treatment [89]. The presence of a good immune infiltrate, such as tumor-infiltrating lymphocytes (TILs), correlates with a good prognosis in a large spectrum of solid tumors, including GI cancers [90,91]. However, pro-tumor immune populations, including TAM2, MDSCs, neutrophil N2, and Tregs, are highly present in these tumors [92,93,94]. Each one contributes to tumor aggressiveness through the secretion of inflammatory cytokines and chemokines, key effector molecules such as MMPs, prostaglandin E2 (PGE2), and TGF-β. The production of these molecules in the TME, as already described, is responsible for EMT and the promotion of tumor migration and invasiveness [95,96].

TAMs play an important role in tumor progression by promoting pro-angiogenic and immunosuppressive signaling [97]. Through the production of chemokines such as CCL17, CCL22, and CCL24, they lead to the recruitment of T helper 2 cells, Tregs, eosinophils, and basophils, inducing an immunosuppressive environment [98,99]. In addition, TAM2s produce inflammatory cytokines and TGF-β, which are associated with a more invasive tumor profile [99]. For these reasons, in GI tumors, especially in CRC, GIST, and HCC, they have a pro-tumor and pro-metastatic action, are associated with poor prognosis, and correlate with worse overall survival [100,101].

MDSCs play an important role in suppressing the immune response through a series of secretory factors such as arginases, nitrites, reactive oxygen species (ROS), immunosuppressive cytokines, and the expansion of immunosuppressive cells such as Tregs [102]. In GI tumors, MDSCs are strongly involved in the regulation of the immune system and act to dampen its response to tumors, promoting the escape of tumor cells from immunosurveillance and increasing both metastasis and recurrence [102]. For this reason, they are associated with poor prognosis and low survival [103]. MDSCs induce immunosuppressive effects through the production of several cytokines, such as IL-6, IL-10, PGE2, and TGF-β [104,105,106]. In addition, MDSCs promote tumor progression through the induction of MMP-9 and TGF-β, which are responsible for the establishment of a more invasive tumor phenotype [105].

Tumor-associated neutrophils (TANs) play a controversial role in tumor progression. Although they are known to have antitumor activity, many studies have shown that the presence of TANs is associated with the promotion of tumor metastatic potential and poor prognosis in many tumor types [107,108]. Especially, type 2 TANs have immunosuppressive action characterized by the production of chemokines, some having a role in cancer cell migration, such as CCL2, CCL3, CCL4, CCL8, CXCL8, and CXCL16 [109]. In HCC, TAN2 promotes tumor progression and metastasis by inducing the recruitment of TAM2 and Tregs into the TME [109].

Tregs are essential for the maintenance of immunological homeostasis and self-tolerance. In tumors, they may have extensive suppressive activity, secreting immunomodulatory cytokines and cytolytic molecules that allow them to regulate immune responses [110]. In GI tumors, high numbers of Tregs are often associated with poor prognosis and low survival rates [111,112]. It has been extensively studied that Tregs generate a strong pro-inflammatory environment in GI tumors through the secretion of inflammatory cytokines such as IL-12, TGF-β, and TNF-α promoting an immunosuppressive TME and, therefore, supporting tumor invasiveness and metastasis formation [113,114].

## 4. miRNA in Gastrointestinal Cancer

MicroRNAs (miRNAs) are small, endogenous, non-coding RNAs, 17–25 nucleotides long that regulate gene-expression post-transcriptionally by recognizing complementary sites in the 3′untraslated region (UTR) of the target messenger RNA (mRNA). A single miRNA is able to target hundreds of mRNAs and influence the expression of many genes. A disruption of the miRNA-mediated gene expression control may lead to environmental stresses usually implicated in the development and progression of human cancer, such as starvation, hypoxia, oxidative stress, and DNA damage [115]. As a matter of fact, miRNAs are involved in cancer initiation, progression, and metastasis. Indeed, many miRNAs are found to be up- or down-regulated in cancer samples when compared to normal tissue (Table 1). miRNAs can modulate the expression of mechanisms such as toll-like receptors, Wnt/β-catenin, Hedgehog, and Jak/Stat signaling pathways [116]. Of note, they can act as oncogenes or tumor suppressors through various mechanisms [117].

## 5. miRNA and Tumor Microenvironment

As previously described, TME is a key factor in the progression and proliferation of tumor cells and drug resistance [139]. Recently, attention was given to the role of miRNAs in the modulation of different types of cells in the TME, such as immune cells and CAFs [139,140,141]. Aberrant expression of miRNAs leads to the reprogramming of cells in CAFs in HCC and GC. In detail, miR-21 converts hepatic stellate cells in activated CAFs by regulating TGF-β signaling and inducing phosphatase and tensin homolog (PTEN) down-regulation and consequent up-regulation of the PI3K/Akt signaling pathway in HCC [127,142]. miR-1247-3p converts normal fibroblast in CAFs promoting HCC progression [143]. miR-27a acts as an oncogene inducing the reprogramming of normal fibroblast in CAFs promoting cancer proliferation and metastasis in GC [130]. Moreover, miR-106b directly targets the PTEN gene in CAFs promoting proliferation, migration, and invasion of GC cells [144].

CAFs-derived miR-493-5p plays a role in the progression and cell growth in CCA. In fact, miR-493-5p expression levels are higher in extracellular vesicles derived from CAFs than the ones from normal fibroblasts [141]. miRNAs secreted by CAFs also play a role in drug resistance. For example, GC cells’ resistance to cisplatin treatment is due to the expression of miR-522 derived from CAFs [145]. miRNAs secreted by CAFs also play an important role in CRC. Jiang et al. highlighted the correlation between the expression of exosomal miR-181b-3p derived from CAFs and CRC development and progression [146]. A recent study by Liu et al. demonstrated that exosomal miR-29a targets and reduces proteins expressed on the vascular endothelial cells in CRC, facilitating metastasis [147].

Like CAFs, the activation of TAMs also plays a role in the modulation of TME. In GC, exosomal miR-21 derived from TAMs leads to resistance to cisplatin through suppression of apoptosis and activation of the PI3K/Akt signaling pathway in cancer cells [148]. Other miRNAs promote cancer progression and metastasis regulating immune cell responses, such as miR-192-5p, which modulates tumor-infiltrating Tregs in GC activity [149].

## 6. miRNAs as Potential Biomarkers in Gastrointestinal Cancers

As mentioned above, miRNA levels are altered in different pathological processes of several tumors, including GI cancers. This deregulation may act as a specific tumor signature and could be useful in differential diagnosis and in correlation with specific clinical characteristics. miRNA expression levels could be associated with a better or worse prognosis. For example, miR-451 is associated with a worse prognosis in GC and CRC [150]. Therefore, the idea of using miRNAs as potential biomarkers for GI tumor screening, prognosis, diagnosis, and disease monitoring is increasingly dominant [151,152]. Profiling of miRNAs has advantages over mRNA and protein, such as their stability in body fluids and human formalin-fixed paraffin-embedded (FFPE) tissues, their small size, and their regulatory function on different target molecules and pathways [153].

Moreover, miRNA expression profiles could be analyzed with miRNA microarray platforms, quantitative reverse transcription-polymerase chain reaction (qRT-PCR), or Next-Generation Sequencing (NGS) approaches (such as RNA-seq analysis) [151].

### 6.1. miRNAs as a Biomarker in Gastric Cancer

Usually, oncogenic miRNAs are up-regulated in GC, while tumor-suppressor miRNAs are down-regulated [151]. An alteration of miRNA expression can induce changes in cell proliferation, cell cycle progression, apoptosis, cell migration, and invasion that may lead to GC (Table 2). Several studies demonstrated that miRNAs are able to promote migration and invasion of cancer cells. Hu et al. studied the over-expression of miR-532 in GC tissues and cells compared to normal stomach tissue and surrounding non-cancer tissue [154]. Wound healing and transwell assay demonstrated that miR-532 induces cell migration and invasion in the GC by targeting Nkd1 and inhibiting the Wnt/β-catenin pathway [154]. Numerous miRNAs affect cell migration and invasion in GC, such as miR-215, targeting FOXO1 and up-regulating activated leukocyte cell adhesion molecule (ALCAM) [151] or miR-186, which modulates Twist1 (Table 2) [155,156]. miR-192-5p expression facilitates cell proliferation, the EMT process, and cell invasion in GC by regulating Wnt, TGF-β, and PI3K/Akt signaling pathways [157,158]. Shayimu et al. studied the role of miR-922 on normal gastric epithelial and GC cell lines. This study highlighted the capability of miR-922 to induce cell invasion and migration by promoting MMP-2 and MMP-9 while suppressing SOCS1 expression [138]. Moreover, miR-922 is able to block apoptosis and promote cell proliferation in GC cell lines [138]. The capability of miR-210 to induce, alone or in synergy with long non-coding RNA MIR210HG, cell migration and metastasis was also studied [137]. In particular, c-myc activates miR-210 and MIR210HG, resulting in the induction of cell migration by promoting MMPs [137]. As noted by Zhang et al., miR-21 has an important role in tumor invasion and metastasis in GC. It is up-regulated in GC, and its main target is RECK, a protein involved in the modulation of MMP-2, MMP-9, and MMP-14 expression [159]. Moreover, miR-21 modulates PTEN and Programmed Cell Death 4 (PDCD4) expression, supporting invasion, migration, EMT, and metastasis. These processes are also promoted by the modulation of several EMT proteins, such as vimentin, SNAIL, ACTA2, and TWIST1 [160]. Other studies reported that miR-196a/b over-expression increases radixin (RDX) protein levels and down-regulates MAX dimerization protein 1 (MDX1), promoting cell migration, invasion, and metastasis [161,162]. Other miRNAs, such as miR-370, interfere with the TGF-β signaling pathway, involved in cell migration, and with the Ubiquinol–cytochrome c reductase core protein 2 (UQCRC2) axis regulating EMT [163,164]. On the other side, decreased expression of tumor suppressor miRNAs, such as miR-218 and let-7, promote tumor invasiveness in GC by eliminating repression of the Robo1 pathway and increasing high mobility group AT-hook 2 (HGMB2) expression, respectively [165,166]. In addition, miR-200 family members increase EMT by reducing the expression of E-cadherin repressors [167]. The down-regulation of miR-335 and miR-153 promotes invasion and metastasis processes targeting BCL-w and SNAIL [168,169]. Furthermore, some miRNAs, such as miR-9, miR-10b, and miR-223, have a dual role in GC progression, acting as both tumor promoters and suppressors according to their target genes [151]. Table 2 lists the principal miRNAs that are deregulated in GC.

### 6.2. miRNAs as Biomarker in Cholangiocarcinoma

The aberrant expression of several miRNAs (Table 3) modulates the expression of genes involved in the processes of invasion and cell proliferation in CCA. The up-regulation of miR-10a-5p induces proliferation in CCA cell lines by targeting and modifying PTEN and phospho-Akt (ser473) expression [125]. Zhang et al. also demonstrated that the aberrant expression of miR-30a-5p can inhibit CCA cell apoptosis and promote CCA progression by targeting the SOCS3 gene [189]. In 2018, Wan et al. showed that CCA tissues presented an elevated expression of miR-383 compared to normal hepatic tissue samples [190]. Wan et al. demonstrated that the over-expression of miR-383 negatively regulates the interferon regulatory factor-1 (IRF1), involved in the cell cycle, inhibiting its tumor suppressor role and inducing cell proliferation and migration [190]. It was demonstrated that several miRNAs were identified as prognostic and diagnostic biomarkers in CCA. For instance, Kishimoto et al. demonstrated miR-21 over-expression, thus indicating that it may be used as a biomarker able to distinguish cancer patients from healthy ones. Notably, miR-21 targets NAD(+)-linked 15-hydroxyprostaglandin dehydrogenase (15-PGDH/HPGD), promoting cell growth and up-regulates EMT-related KLF4, N-cadherin, vimentin, Akt and ERK1/2, inducing EMT and invasion [191,192,193]. In addition, the over-expression of miR-27a promotes cell proliferation, migration, and invasion. Effectively, high levels of miR-27a are associated with lymph node metastasis and a poor prognosis in CCA patients [194]. Many studies reported an abnormal expression of miR-29b in several human cancers. miR-29b was down-regulated and associated with poor overall survival in CCA cells and tissues [195]. Proliferation tests, together with flow cytometry analysis, demonstrated that miR-29b influences both cell cycle and apoptosis in CCA cell lines [195].

Sheng et al. reported the up-regulation of Yes-associated protein 1 (YAP1), a transcriptional coactivator of the tumor-suppressive Hippo pathway, and implicated in CCA pathogenesis. Moreover, it was discovered that YAP is the target of miR-16, which results in suppressed CCA determining proliferation, invasion, and metastasis [196].

However, it was observed that seven miRNAs, miR-21, miR-26, miR-106a, miR-150, miR-192, and miR-194, could be employed for differential diagnosis to distinguish patients with CCA from controls [197]. Furthermore, it was discovered that the 4-miRNA CCA signature (miR-30a, miR-200c, miR-141, and miR-425) could differentiate CCA from other GI tumors [198] (Table 4).

**Table 3:** Aberrantly expressed miRNAs in Cholangiocarcinoma, with their specific targets.

miRNA	Target	Effects	Refs.
let-7c	EZH2	Promotes cell migration and invasion Metastasis	[122]
miR-10a-5p	PTEN	Induces cell proliferation	[125]
miR-16	YAP1	Induces cell proliferation Promotes cell invasion Metastasis	[196]
miR-21	KLF4 N-Cadherin VIMENTIN AKT ERK1/2 15-PGDH	Induces cell proliferation and EMT Promotes cell invasion Metastasis	[191,192,193]
miR-23	DNM3	Induces cell proliferation	[199]
miR-27a	D1 CYCLIN E-Cadherin KRAS YAP	Induces cell proliferation Promotes cell migration and invasion Metastasis	[194]
miR-29b	DNMT3B	Influences cell cycle and apoptosis	[195]
miR-30a-5p	SOCS3	Inhibits apoptosis Induces cell proliferation	[189]
miR-96	MTSS1	Induces cell proliferation Metastasis	[200]
miR-137	WNT2B	Regulates apoptosis Induces cell migration and invasion	[201]
miR-181b-5p	PARK2	Promotes cell migration	[202]
miR-196	HAND1	Promotes cell growth Metastasis	[203]
miR-320	VEGFR2 NRP-1	Induces cell growth and proliferation Metastasis	[204]
miR-383	IRF1	Induces cell proliferation Promotes cell migration	[190]
miR-424-5p	ARK5	Promotes cell migration and invasion Induces EMT	[205]

**Table 4:** Up- or down-regulated miRNAs employed in differential diagnosis in Cholangiocarcinoma.

miRNA	Up/Down-Regulated	Ref.
miR-21	Up-regulated	[206]
miR-26	Up-regulated	[206]
miR-30a	Up-regulated	[198]
miR-106a	Down-regulated	[207]
miR-141	Down-regulated	[198]
miR-150	Up-regulated	[206]
miR-192	Up-regulated	[208]
miR-194	Up-regulated	[209]
miR-200c	Down-regulated	[198]
miR-425	Down-regulated	[198]

### 6.3. miRNAs as Biomarkers in Hepatocellular Carcinoma

Recently, numerous studies have analyzed the role of miRNAs in the development and progression of HCC [210]. miRNA profiles have proven that they are an important source for potential biomarkers as they made possible the distinction of liver cancer cells from hepatocytes [144] (Table 5). The WNT/β-catenin pathway is frequently up-regulated, and it is involved in the proliferation, migration, invasion, and survival of liver cancer cells. Moreover, it was discovered that several miRNAs implicated in this deregulation, including miR-21, miR-106b, miR-135, and miR-315, are overexpressed, and miR-122, miR-145, miR-214 are down-regulated [144]. Additionally, the down-regulation of miR-221/-222, miR-30, and miR-148a contribute to the E-cadherin loss, reducing cell–cell adhesion and promoting EMT [211]. Down-regulation of miR-23b and miR-34a plays a role in HCC progression by inducing cell proliferation, migration, invasion, and metastasis [212].

It has been demonstrated that down-regulation of different miRNAs, such as miR-199a-5p or miR-29c, promotes tumorigenesis and increases cell invasion in HCC by modulating MMP activities and regulating the cell cycle, respectively [213,214]. Instead, miR-9 over-expression promoted migration and invasion in HCC cells, likewise in other types of GI cancer such as colon cancer and GC [123,124,215,216]. Sun et al. studied the connection between the over-expression of miR-1246 and the putative target CADM1, involved in cell–cell interaction, and the capacity of the miRNA to induce metastasis in HCC [217]. Various miRNAs, in addition, enhance HCC progression mediating the EMT event, such as miR-330-3p and miR-192 [218].

About nineteen deregulated miRNAs are implicated in angiogenesis, invasion, and metastasis, such as miR-122, which enhance these processes by inhibiting the p53 signaling pathway and modulating disintegrin and metalloprotease 17 (ADAM17) [219]. miR-139 and miR-151 were also involved in the ADAM17 pathway facilitating angiogenesis, invasion, and metastasis [220].

Aberrant miRNA expression profiles between liver cancer and normal liver tissues have been identified. For instance, Nagy et al. identified several overexpressed miRNAs, such as miR-421, miR-183, miR-182, miR-96, and miR-301, in liver cancer patients. At the same time, they also identified a number of down-regulated miRNAs, including miR-195, miR-139, miR-326, and miR-145 [221].

**Table 5:** Aberrantly expressed miRNAs in Hepatocellular carcinoma, with their specific targets.

miRNA	Target	Effects	Refs.
miR-9	KLF17	Promotes cell migration and invasion	[123]
miR-21	PTEN PDCD4	Metastasis	[222]
miR-23b	uPA MET	Promotes cell proliferation Induces cell invasion	[212,223]
miR-29c	RPS15A	Promotes cell invasion Regulates cell cycle	[214]
miR-30	SNAIL	Induces EMT	[211]
miR-34a	HDAC1 D1 CYCLIN CDK2/4 FOXMI BCL-2	Induces cell proliferation Promotes cell invasion and migration Drug resistance	[212,224,225,226]
miR-96	SOX6	Induces cell proliferation Promotes cell migration and invasion	[227]
miR-106b	PTEN	Induces cell proliferation Promotes cell migration and invasion	[228]
miR-122	ADAM17 WNT1 TACE LMNB2	Induces cell proliferation Promotes cell invasion Angiogenesis and metastasis	[219,229,230]
miR-124-3p	CRKL	Promotes cell migration and invasion Metastasis	[231]
miR-130b	Notch-Dll1	Promotes cell migration and invasion	[232]
miR-135	APC AXIN	Metastasis	[222]
miR-139	ADAM17 ROCK2	Induces cell proliferation Metastasis	[229]
miR-144	FOXK1	Modulates glycolysis	[233]
miR-145	IRS1 IRS2 OCT4 β-Catenin IGF-IR	Induces cell proliferation Promotes cell migration and invasion	[234]
miR-148a	c-MET HRIP c-MYC WNT1 SNAIL1 DNMT1	Induces EMT Metastasis	[211]
miR-151	ADAM17 RHOGDIA	Promotes cell invasion Angiogenesis and metastasis	[235]
miR-182	FOXO3a MTSSI pRB CEPBA RASA1 c-MYC	Induces cell proliferation Angiogenesis and metastasis	[236,237,238]
miR-183	SOCS6	Induces cell proliferation Promotes cell invasion	[239]
miR-185	AKT1	Induces cell proliferation	[240]
miR-195	CDK6 CYCLIN D1 YAP WNT3a VEGF	Regulates cell cycle and apoptosis Induces EMT Angiogenesis and metastasis	[241,242]
miR-199	DDR1 mTOR c-Met	Promotes cell invasion Regulates cell cycle Drug resistance	[213,243]
miR-214	HDGF β-Catenin	Angiogenesis	[244]
miR-221/-222	PTEN E-cadherin	Induces EMT	[211]
miR-301	GAX	Metastasis	[222]
miR-315	APC Axin	Metastasis	[222]
miR-326	LASP1 RAB21	Induces cell proliferation Promotes cell invasion	[245]
miR-330-3p	EREG	Regulates EMT	[218]
miR-409	JAK2 STAT3	Inhibits apoptosis Induces cell proliferation Promotes cell viability	[246]
miR-421	SOX9 PTEN MMP-3	Induces cell proliferation and EMT Promotes cell invasion	[247,248,249]
miR-520c-3p	PTEN	Promotes cell migration and invasion	[250]
miR-539	MAP2K1	Promotes cell migration and invasion Induce cell proliferation Inhibits apoptosis	[251]
miR-579-3p	PIK3CA	Tumor development	[252]
miR-1246	CADM1	Metastasis	[217]
miR-4521	FAM129A	Regulates cell growth and apoptosis	[253]

### 6.4. miRNAs as Biomarkers in Colorectal Cancer

As mentioned above, CRC cells are characterized by a high capability of proliferation, invasion, and metastasis. Among the factors that are involved in CRC development, epigenetic modifications have been reported. CRC pathogenesis is characterized by a stage-specific miRNA expression. Table 6 lists miRNAs involved in the development and progression of CRC. Let-7, miR-21, miR-29a, and cluster miR-17-92 are relevant [254]. Let-7 is deregulated in several GI cancers and modulates Ras and Myc expression controlling tumor progression and metastasis in CRC [255]. The increased expression level of miR-21 is associated with invasion and liver and lymph node metastasis [256]. miR-29a is a metastasis-promoter due to its tumor suppressor target gene Kruppel-like factor 4 (KLF4) that up-regulates MMP-2 expression and, at the same time, down-regulates E-cadherin expression [257]. Cluster miR-17-92 is typically amplified and has several target genes involved in CRC progression and metastasis. Especially, miR-17 targets the MYC family, increasing tumor progression and invasion [258,259]. Sun et al. demonstrated the role of miR-103a-3p in the promotion of cell invasion and metastasis by regulating the glycolysis process mediated by the Hippo pathway [134]. MiR-152-3p also promotes cell adhesion and metastases. In particular, miR-152-3p negatively regulates the Aquaporin-11 (AQP11) protein, which is usually involved in the repression of cell growth and adhesion [135]. Moreover, Zhu et al. suggested that inhibition of miR-152-3p could stop the progression of CRC [135]. The aberrant expression of miR-23a-3p is also involved in CRC development. This miRNA, in fact, inhibits the expression of NDRG4 in cancer cells with the consequent increase in cell proliferation, migration, and invasion [129]. miRNAs are also implicated in drug resistance phenomena. It has been demonstrated that miR-93-5p is up-regulated in CRC cells that are resistant to chemotherapeutics and may regulate proteins involved in multidrug resistance and target cyclin-dependent kinase inhibitors [133]. Additionally, miR-101 was reported as a tumor suppressor that is down-regulated in CRC, and its direct target is Cyclooxygenase-2 (COX-2), whose high expression contributes to cell growth and invasion [153].

The over-expression of miR-125b promotes invasion and EMT, enhancing CXCR4 expression; in turn, the CXCL12/CXCR4 axis induces miR-125b expression. Moreover, miR-125b down-regulates the p53 pathway, promoting cell proliferation [260].

Recent studies have demonstrated that the tumor suppressor miR-137-3p is down-regulated in several cancers, such as non-small cell lung cancer (NSCLC), liver cancer, breast cancer, and CRC. miR-137-3p promotes migration, EMT, and invasion of CRC cells in a lysine-specific demethylase 1 (LDS-1)-dependent manner [260].

In addition, miR-106b up-regulation determines the enhancement of proliferation, invasion, and migration in CRC cells via PTEN modulation [144].

miR-145-5p targets cell cycle-associated protein-3 (CDCA3), determining cell growth and EMT suppression [261].

Finally, Volker et al. investigated miR-192, miR-17, and miR-200c down-regulation in CRC invasion and metastatic process. These three miRNAs negatively control target genes associated with ECM remodeling in fibroblasts. Notably, miR-192 targets integrins, such as ITGB1 and ITGAV, inhibiting cell adhesion and metastasis; miR-200c modulates the EMT process via E-cadherin repressors, ZEB1 and ZEB2 [259]. In fact, in this study, it was shown that the enhancement of miR-192, miR-17, and miR-200c suppresses the invasiveness and metastasis of CRC cells.

**Table 6:** Aberrantly expressed miRNAs in Colorectal cancer, with their specific targets.

miRNA	Target	Effects	Refs.
let-7	RAS MYC	Induces cell progression Metastasis	[255]
miR-9	E-Cadherin	Promotes cell migration and invasion	[215]
miR-17	P130	Induces cell progression	[262]
miR-17-92 cluster	C-MYC E2F	Induces cell progression Promotes cell invasion	[258,259]
miR-20a-5p	SMAD4	Promotes cell invasion and migration Metastasis	[263]
miR-21	TNF-α PDCD4 RECK PTEN	Promotes cell migration Induces cell proliferation and EMT Metastasis	[256,264]
miR-23a-3p	NDRG4	Induces cell proliferation Promotes cell migration and invasion	[129]
miR-29a	KLF4 MMP-2	Induces EMT Metastasis	[257]
miR-31	FIH1	Promotes cell invasion and migration Induces cell proliferation	[265]
miR-34	TP53	Induces cancer progression	[266]
miR-93-5p	CDK inhibitor	Drug resistance	[133]
miR-101	COX-2 ZEB1 EZH2	Induces cell proliferation and EMT Promotes cell invasion	[153]
miR-103a-3p	Hyppo	Promotes cell invasion Metastasis	[134]
miR-106b	PTEN	Induces cell proliferation Promotes cell migration and invasion	[144]
miR-125b	BAK1 BMF CXCR4	Induces cell proliferation and EMT Promotes cell invasion	[260]
miR-126	CXCR4	Metastasis	[267]
miR-135	APC	Promotes tumorigenesis	[268]
miR-137-3p	LDS-1	Induces EMT Promotes cell migration and invasion	[260]
miR-145-5p	CDCA3	Induces EMT Promotes cell invasion	[261]
miR-148a	MMP7	Promotes cell invasion	[269]
miR-152-3p	AQP11	Metastasis	[135]
miR-192	ITGB1 ITGAV	Promotes cell invasion Metastasis	[259]
miR-200c	SOX2 ZEB1 ZEB2	Promotes cell invasion and EMT	[259,270]
miR-483	EI24	Induces cell proliferation Promotes cell invasion Metastasis	[271]

### 6.5. miRNAs as Biomarkers in Gastrointestinal Stromal Tumors

A growing number of research has shown that miRNAs play an important role not only in tumorigenesis but also in the risk stratification of GIST [272]. Table 7 briefly summarizes miRNAs of major significance in invasion, EMT, and metastasis in GIST.

miR-196a is one of the most relevant miRNAs involved in tumor progression. In fact, a positive correlation was found between the over-expression of miR-196a and tumor progression from metaplasia to adenocarcinoma [272]. In particular, miR-196a up-regulates annexin A1 (ANXA1), involved in the invasion process, increasing the risk of metastasis formation [273]. miR-186 inhibition is associated with the over-expression of genes implicated in GIST metastasis by modulating the Met/Akt signaling pathway [274]. Therefore, miR-196a over-expression and low expression of miR-186 are associated with poorer prognosis in GIST patients [47].

Yamamoto et al. demonstrated that the down-regulation of miR-133b determines the over-expression of Fascin-1, an actin-binding protein important in the regulation of cell adhesion and migration. In addition, they discovered the correlation between over-expression of Fascin-1 and worse prognosis in GIST patients. For this reason, Fascin-1 should be a useful biomarker to predict cancer aggressiveness [275].

It was found that the down-regulation of miR-137 can control EMT in GIST via TWIST1 inhibition [276]. Liu et al. demonstrated that miR-152 plays a tumor suppressor role by targeting genes associated with cell proliferation, migration, and invasion, and it results in down-regulated in GIST [277]. Recently, a study on miR-218 showed that its down-regulation inhibits cell proliferation, migration, and invasion through direct targeting of the KIT gene [278]. Many different tumors exhibit a down-regulation of let-7 family members, which is also implied in GIST migration, invasion, and metastasis [272].

Gyvyte et al. investigated two deregulated miRNAs in GIST: miR-375-3p and miR-200b-3p. Their down-regulation increases cell viability and migration through the modulation of different target gene expressions, such as KIT, EGFR, ETV1, and the JAK/STAT3 pathway [279].

**Table 7:** Aberrantly expressed miRNAs in Gastrointestinal Stromal cancer, with their specific targets.

miRNA	Target	Effects	Refs.
let-7-c	HOXA1 MMP1 C/EBP-α	Induces cell proliferation Promotes cell migration and invasion	[272]
miR-133b	FSCN1	Enhance cell proliferation Promotes cell invasion	[276]
miR-137	TWIST1	Induces cell cycle arrest Promotes cell migration and EMT Apoptosis	[276]
miR-152	CTSL	Induces cell proliferation Promotes cell migration and invasion	[277]
miR-186	IGFBP3 AKT HGFR CXCR4 EFEMP1	Promotes cell migration and invasion Metastasis	[274]
miR-196a	ANXA1	Promotes cell invasion	[272,273]
miR-218	AKT KIT	Induces cell proliferation Promotes cell migration and invasion	[276,278]
miR-200b-3	EGFR ETV1 STAT1	Induces cell proliferation Promotes cell migration and invasion	[279]
miR-375-3p	KIT PDGFRA JAK2	Induces cell proliferation Promotes cell migration and invasion	[279]

## 7. Other Biomarkers in Gastrointestinal Cancers

In addition to the use of miRNAs as possible biomarkers, several molecules have emerged over the years as biomarkers helpful for the diagnosis, follow-up, and response to treatment in GI cancers. To date, the most widely used biomarker in the clinic for GI cancer is carcinoembryonic antigen (CEA), a protein found in CRC and CCA patients that plays an important role in cell adhesion and intracellular signaling [280]. It is used as a prognostic, diagnostic, and response-to-therapy biomarker and as a target for cancer immunotherapy (DOTAP) [281]. Indeed, high levels of CEA in the blood of patients with CRC are associated with disease progression, while a decrease is found in patients after surgery [282]. Moreover, tumor-associated antigens, such as CA19-9, a marker in the diagnosis and follow-up of some GI cancers such as pancreatic cancer, CRC, GC, and CCA [283,284,285], are extensively used. The first is found at high levels in the serum of CRC patients, while the second is considered the first tumor marker for GC [286,287,288]. Alpha-fetoprotein (AFP) is a glycoprotein capable of binding to different types of membrane receptors and intracytoplasmatic proteins, blocking or enhancing the responses of intracellular signaling pathways. It is an important biomarker used for early diagnosis and prognosis of HCC patients [289].

## 8. Nutraceuticals

Nowadays, conventional anticancer therapy implicates the use of radiotherapy and chemotherapy, but such treatments are still expensive and inefficient, especially due to drug resistance and severe adverse events. To date, there are many experimental studies showing the usefulness of nutraceuticals as a complementary approach to standard therapy, thanks to their low toxicity and multiple biological activities [290,291]. Nutraceuticals are also known as bioactive compounds isolated from plants or food and include dietary fibers, antioxidants, phytochemicals, polyunsaturated fatty acids, amino acids, prebiotics and probiotics, and other types of natural food [292].

Many of them have multiple therapeutic properties, such as antioxidant, anti-inflammatory and anticancer, and they seem to be very interesting in the management or treatment of malignancies, cardiovascular diseases, diabetes, obesity, osteoporosis, and the immune system, used alone or in combination with other drugs [293].

Much evidence has revealed that numerous amounts of bioactive substances or extracts from medical plants play an important role in the treatment of different types of GI tumors [290].

Cairicoside E (CE) is a natural herbal medical compound isolated from Ipomoea Cairica (Convolvulaceae). CE modulates the EMT through the down-regulation of aquaporine-5 (AQP5), determining an anti-metastatic effect [294].

Berberine (BBR) is an alkaloid used in Chinese plant-based medicines. It has a wide spectrum of therapeutic actions, such as inhibition of cell proliferation, migration, induction of cellular death, and the enhancement of chemosensitivity through the modulation of NF-κB, PI3K/Akt, and the MAPK signaling pathways. In addition, it is involved in immunity, inflammation [295,296], and, in some cases, in reversing gastric cancerogenesis [297].

Particularly, BBR up-regulates E-cadherin expression and down-regulates N-cadherin, fibronectin, and vimentin expressions to modulate the EMT. Furthermore, BBR suppresses migration and invasion of tumor cells via the IL-6/JAK2/STAT3 signaling pathway and inhibiting MMP-9 protein levels.

BBR up-regulates miR-203 expression, a tumor-suppressive miRNA that binds at the 3′-UTR of Bcl-w oncogene, also resulting in a decrease in chemoresistance [298].

Oleanolic acid (OA) is a pentacyclic triterpene isolated from several plants, including Olea europaea [299]. An antitumoral effect has been demonstrated through the over-expression of miR-98-5p involved in the regulation of Treg/Th17 balance in GC tissues [300].

Paeoniflorin is a bioactive substance of Radix Paeoniae Rubra and is potentially used as a novel therapeutic agent in GC TME. In fact, it improves the immune microenvironment through the up-regulation of miR-149 in gastric CAFs, inhibiting the secretion of IL-6 and leading to the inactivation of the IL-6/STAT3/MMP signaling axis [301].

Sulforaphane is an isothiocyanate derived from the *Brassicaceae* family, including cabbage and broccoli. It has several activities, including chemopreventive and chemotherapeutic, in different tumors such as lung, bladder, and CRC. As an anticancer drug, Sulforaphane affects all three stages of carcinogenesis. Many studies revealed that it is able to suppress progression and angiogenesis processes in CRC by the inhibition of HIF-1α and VEGF expression [302,303].

Resveratrol (RV) is a polyphenol found in red wine, grapes, peanuts, and other food products that has been reported to have antineoplastic activity on different malignancies [304]. It could present an antitumor potentiality in CCA. In fact, RV can inhibit the secretion of IL-6 from CAFs, preventing the induction of EMT and cancer cell migration. Furthermore, it strongly promotes E-cadherin expression while suppressing N-cadherin, thus, resulting in a reverse phenomenon of the EMT and a reduction in invasiveness and metastasis in cancer cells [305].

Curcumin (CUR) is a non-flavonoid polyphenol purified from the rhizome of the *Curcuma longa*. It is a pleiotropic molecule with anti-inflammatory, antioxidant, immunomodulatory, and antimicrobial properties [306]. Recent studies have shown that CUR has an antitumor effect on several malignancies, including GI cancers [307].

CUR is a multitarget drug capable of decreasing the expression of molecules involved in angiogenesis, such as VEGF, and tumor invasion, such as intracellular adhesion molecule-1 (ICAM-1), MMP-2, and MMP-9 in CCA [308].

Furthermore, CUR can suppress proliferation, migration, and invasion processes promoting apoptosis of cancer cells in HCC by targeting the circ_0078710/miR-378b/PRIM2 signaling pathway [309] and up-regulating the miR-200 family, involved in the EMT suppression [310].

CUR down-regulates miR-21 expression in CRC and GC. As mentioned above, miR-21 plays an important role in cancer cell migration and invasion through the activation of the PI3K/Akt/mTOR pathway and modulation of MMP-2, MMP-9, and MMP-14 [159,310].

Several plant- and food-derived compounds have a high potential to treat GI tumors in line with relatively low toxicity to normal cells. Indeed, they could be used in combination with conventional anticancer drugs in order to have a potential synergistic effect in cancer therapy [295,311].

## 9. Conclusions and Perspective

In summary, this review systematically describes aspects related to the mechanisms responsible for tumor migration and invasiveness in GI cancers. Metastasis is the leading cause of death from GI cancer, so it is of paramount importance to study the mechanisms that regulate tumor migration, invasion, and metastasis to propose novel therapeutic targets and biomarkers. The migration of tumor cells and their ability to form metastases are regulated by mechanisms driven by the TME. In GI tumors, the TME is composed of different cell types which cooperate to promote and trigger metastasis formation. Specifically, CAFs play a pivotal role in tumor progression by enhancing EMT and, thus, promoting migration and metastasis formation. These processes are further supported by the presence of an immunosuppressive infiltrate, mainly composed of Treg, MDSC, and TAM2 cells, and by the presence of chemokines and cytokines such as CXCL12, IL-6, and TGF- β.

Recent studies have also demonstrated the critical role of miRNAs in promoting tumor progression and metastasis formation in GI tumors. They may modulate signaling pathways involved in migration and tumor invasion, such as Wnt/β-catenin, Hedgehog, and Jak/Stat. Many miRNAs are found to be up- or down-regulated in tumor tissues compared to healthy tissues, and to date, they are being considered as potential predictors for prognosis, monitoring, and diagnosis of GI cancer patients. Of note, recent studies have shown that the use of nutraceuticals, bioactive compounds isolated from plants or foods, would be useful in GI cancers as a complementary approach to standard therapy. Indeed, they are able to target miRNAs and other molecules that regulate several mechanisms involved in tumor migration and invasiveness. This could pave the way for new targeted therapies for GI cancer treatment.

The high aggressiveness and late diagnosis in patients with GI tumors often result in a poor prognosis. Therefore, it is necessary to develop new tools to improve early diagnosis and prognosis for these patients. A growing number of studies are highlighting the important role of miRNAs as potential markers in many neoplasms. The use of miRNAs as biomarkers offers the great advantage of developing minimally invasive methods due to their stability, which renders them easy to detect in different body fluids, such as blood.

On the other hand, the main disadvantage of their use in large-scale therapeutic applications could be the difficulty of predicting the overall effect of the miRNAs of interest due to the possible high number of target genes. Especially in a therapeutic context, their application could represent a risk because of the occurrence of unknown and unpredictable side effects, possibly triggering physiological function disorders or the development of additional diseases. For this reason, Zhang et al. proposed the design of new delivery systems for miRNA that recognize specific features at the site of the malignant lesion [312].

Further investigations are, therefore, needed for the application of miRNAs as tumor biomarkers. In fact, they could offer important opportunities for the development of new diagnostic and prognostic strategies that would help to improve the clinical outcome of patients with GI tumors.

## Figures and Tables

**Table 1:** Up- or down-regulated miRNAs in various gastrointestinal tumors compared to normal tissues.

miRNA	Up/Down-Regulated	Type of Cancer	Ref.
let-7	Up	Colon cancer	[118]
	Down	Hepatocellular carcinoma	[119]
	Down	Gastric cancer	[120]
	Up	Hepatocellular carcinoma	[121]
	Down	Cholangiocarcinoma	[122]
miR-9	Up	Hepatocellular carcinoma	[123]
	Up	Gastric cancer	[124]
miR-10a-5p	Up	Cholangiocarcinoma	[125]
miR-21	Up	Colorectal cancer	[126]
	Up	Hepatocellular carcinoma	[127]
	Up	Cholangiocarcinoma	[128]
miR-23	Up	Hepatocellular carcinoma	[121]
miR-23a-3p	Up	Colorectal cancer	[129]
miR-27a	Up	Gastric cancer	[130]
miR-29s	Down	Cholangiocarcinoma	[131]
miR-31	Down	Colorectal cancer	[132]
miR-93-5p	Up	Colorectal cancer	[133]
miR-103a-3p	Up	Colorectal cancer	[134]
miR-152-3p	Up	Colorectal cancer	[135]
miR-199a-5p	Down	Hepatocellular carcinoma	[136]
miR-210	Up	Gastric cancer	[137]
miR-922	Up	Gastric cancer	[138]

**Table 2:** Aberrantly expressed miRNAs in Gastric cancer, with their specific targets.

miRNA	Target	Effects	Refs.
let-7	RAB40C HMGA2 CCR7	Induces proliferation Promotes invasion	[166]
miR-9	NKFB1 CCND1 CDX2	Induces proliferation Metastasis	[170,171]
miR-10b	HOXD10	Promotes cell migration and invasion Metastasis	[172]
miR-21	RECK PTEN PDCD4 VIMENTIN SNAIL TIMP3	Promotes cell migration Induces EMT Metastasis Drug resistance	[159,160,173]
miR-106b	PTEN RB1 TIMP2	Induces cell proliferation Promotes cell migration and invasion	[174]
miR-107	DICER1	Promotes cell migration and invasion	[175]
miR-124	ROCK1	Induces cell proliferation Promotes cell invasion	[176]
miR-126	CRK PI3KR2	Induces cell proliferation Promotes cell migration and invasion Metastasis	[177,178]
miR-130a	RUNX3	Induces metastasis	[179]
miR-135b	n.d.	n.d.	[180]
miR-148a	CDKN1B	Induces cell proliferation Regulates cell cycle Metastasis	[181]
miR-153	SNAIL	Promotes cell migration and invasion Metastasis	[169]
miR-186	TWIST1	Promotes cell migration	[156]
miR-192-1-3p	PDCD2	Induces cell proliferation	[182]
miR-192-5p	SMG-1	Induces cell proliferation and EMT	[158]
miR-196	RADIXIN MXD1	Promotes cell migration and invasion Metastasis	[161,162]
miR-200	DLC-1 ZEB1 ZEB2 BCL-2 XIAP	Induces cell proliferation and EMT Promotes cell migration and invasion	[167,183]
miR-210	DRD5	Promotes cell migration and invasion	[137]
miR-215	FOXO1	Promotes cell migration	[155]
miR-218	ROBO1	Promotes cell invasion Metastasis	[165]
miR-223	STMN1	Promotes cell invasion Metastasis	[184,185]
miR-324-5p	PTEN	Induces cell proliferation Promotes apoptosis	[186]
miR-335	BCL-w	Metastasis	[168]
miR-370	TGF-β-RII UQCRC2	Induces EMT Metastasis	[163,164]
miR-452	EPB41L3	Promotes cell migration and invasion	[187]
miR-532	NKD1	Promotes cell migration and invasion	[154]
miR-633	n.d.	n.d.	[188]
miR-922	SOCS1	Induces cell invasion Promotes cell migration	[138]

## Data Availability

Not applicable.

## Abbreviations

ALCAM	Activated leukocyte cell adhesion molecule
ANXA1	Annexin A1
AQP5	Aquaporine-5
AQP11	Aquaporine-11
AFP	Alpha-fetoprotein
bFGF	Basic fibroblast growth factor
BBR	Berberine
CE	Cairicoside E
CAFs	Cancer-associated fibroblasts
CEA	Carcinoembryonic antigen
CTSL	Cathepsin L
C/EBP-α	CCAAT Enhancer Binding Protein Alpha
CXCR4	CXC chemokine receptor 4
CDCA3	Cell cycle-associated protein-3
CCA	Cholangiocarcinoma
JNK	c-Jun amino-terminal kinases
CRC	Colorectal cancer
CUR	Curcumin
COX-2	Cyclooxygenase-2
DC	Dendritic cells
ADAM17	Disintegrin and metalloprotease 17
EFEMP1	Epidermal growth factor-containing fibulin-like extracellular matrix protein 1
EGFR	Epidermal growth factor receptor
EMT	Epithelial-mesenchyme transition
ETV1	ETS transcription factor 1
ECM	Extracellular matrix
ERK1/2	Extracellular regulated kinase 1/2
FSCN1	Fascin actin-bundling protein 1
FAP	Fibroblast activation protein
FFPE	Formalin-fixed paraffin-embedded
GC	Gastric cancer
GI	Gastrointestinal
GIST	Gastrointestinal stromal tumor
HCC	Hepatocellular carcinoma
HGFR	Hepatocyte growth factor receptor
HGMB2	High mobility group AT-hook 2
ICIs	Immune checkpoint inhibitors
IDO	Indoleamine-pyrrole2,3-dioxygenase
IGF-1	Insulin-like growth factor-1
IGFBP3	Insulin-like growth factor-binding protein 3
IRF1	Interferon regulatory factor-1
ICC	Interstitial cell of Cajal
ICAM-1	Intracellular adhesion molecule-1
JAK	Janus kinase
KLF4	Kruppel-like factor 4
LDS-1	Lysine-specific demethylase 1
MMP	Matrix metalloproteases
MDX1	MAX dimerisation protein 1
mRNA	Messenger RNA
miRNAs	MicroRNAs
MAPKs	Mitogen-activated protein kinase
MDSCs	Myeloid-derived suppressor cells
15-PGDH/HPGD	NAD(+)-linked 15-hydroxyprostaglandin dehydrogenase
NGS	Next-Generation Sequencing
NSCLC	Non-small cell lung cancer
NF-κB	Nuclear factor kappaB
OA	Oleanolic acid
PTEN	Phosphatase and tensin homolog
PI3K	Phosphatidylinositol 3-kinase
PDGF	Platelet-derived growth factor
PDGFRA	Platelet-derived growth factor receptor A
PDCD4	Programmed Cell Death 4
PGE2	Prostaglandin E2
AKT	Protein kinase B
qRT-PCR	Quantitative reverse transcription-polymerase chain reaction
RDX	Radixin
RKT	Receptor tyrosine kinase
ROS	Reactive oxygen species
RV	Resveratrol
RECK	Reversion Inducing Cysteine Rich Protein with Kazal Motifs
STAT	Signal transducer and activator of transcription
Tregs	T regulatory cells
TGF-β	Transforming growth factor-β
TME	Tumor microenvironment
TAMs	Tumor-associated macrophages
TANs	Tumor-associated neutrophils
TILs	Tumor-infiltrating lymphocytes
TWIST	Twist family bHLH transcription factor 1
KIT	Tyrosine kinase
UQCRC2	Ubiquinol-cytochrome c reductase core protein 2
VEGF	Vascular endothelial growth factor
YAP1	Yes-associated protein 1
α-SMA	α-smooth muscle actin
